# Susceptibility-weighted cardiovascular magnetic resonance in comparison to T2 and T2 star imaging for detection of intramyocardial hemorrhage following acute myocardial infarction at 3 Tesla

**DOI:** 10.1186/s12968-014-0086-9

**Published:** 2014-10-28

**Authors:** Ananth Kidambi, John D Biglands, David M Higgins, David P Ripley, Arshad Zaman, David A Broadbent, Adam K McDiarmid, Peter P Swoboda, Tarique Al Musa, Bara Erhayiem, John P Greenwood, Sven Plein

**Affiliations:** Multidisciplinary Cardiovascular Research Centre & Leeds Institute of Cardiovascular and Metabolic Medicine, University of Leeds, LS2 9JT Leeds, UK; Division of Medical Physics & Multidisciplinary Cardiovascular Research Centre, Leeds Institute of Cardiovascular and Metabolic Medicine, University of Leeds, Leeds, UK; Philips Healthcare, Philips Centre, Guildford, Surrey UK

**Keywords:** Hemorrhage, Magnetic resonance imaging, Cardiovascular magnetic resonance, Myocardial infarction, Susceptibility

## Abstract

**Background:**

Intramyocardial hemorrhage (IMH) identified by cardiovascular magnetic resonance (CMR) is an established prognostic marker following acute myocardial infarction (AMI). Detection of IMH by T2-weighted or T2 star CMR can be limited by long breath hold times and sensitivity to artefacts, especially at 3T. We compared the image quality and diagnostic ability of susceptibility-weighted magnetic resonance imaging (SW MRI) with T2-weighted and T2 star CMR to detect IMH at 3T.

**Methods:**

Forty-nine patients (42 males; mean age 58 years, range 35–76) underwent 3T cardiovascular magnetic resonance (CMR) 2 days following re-perfused AMI. T2-weighted, T2 star and SW MRI images were obtained. Signal and contrast measurements were compared between the three methods and diagnostic accuracy of SW MRI was assessed against T2w images by 2 independent, blinded observers. Image quality was rated on a 4-point scale from 1 (unusable) to 4 (excellent).

**Results:**

Of 49 patients, IMH was detected in 20 (41%) by SW MRI, 21 (43%) by T2-weighted and 17 (34%) by T2 star imaging (p = ns). Compared to T2-weighted imaging, SW MRI had sensitivity of 93% and specificity of 86%. SW MRI had similar inter-observer reliability to T2-weighted imaging (*κ* = 0.90 and *κ* = 0.88 respectively); both had higher reliability than T2 star (*κ* = 0.53). Breath hold times were shorter for SW MRI (4 seconds vs. 16 seconds) with improved image quality rating (3.8 ± 0.4, 3.3 ± 1.0, 2.8 ± 1.1 respectively; p < 0.01).

**Conclusions:**

SW MRI is an accurate and reproducible way to detect IMH at 3T. The technique offers considerably shorter breath hold times than T2-weighted and T2 star imaging, and higher image quality scores.

## Background

The aim of emergency treatment for ST-elevation acute myocardial infarction (AMI) is coronary reperfusion, optimally via primary percutaneous coronary intervention (PCI). In approximately 40% of patients, reperfusion by primary PCI is associated with detectable reperfusion injury [1]. Reperfusion injury may manifest as microvascular obstruction (MO), which is associated with adverse functional outcome [2] and worse prognosis [3,4]. A subset of patients with MO may also have hemorrhage within the infarcted myocardium. Intramyocardial hemorrhage (IMH) is independently associated with adverse prognosis over and above MO alone [5]. The most sensitive clinical way of detecting IMH is by cardiovascular magnetic resonance (CMR) imaging [6]. The breakdown products of hemoglobin within IMH exert a paramagnetic effect which shortens T2 relaxation times, resulting in the presence of a characteristic hypointense infarct core on T2-weighted (T2w) or T2* imaging [7]. However, this important prognostic marker has been relatively underused in the clinical setting. One reason for this underuse is the difficulty in obtaining reliable diagnostic quality images, which for dark-blood T2w or T2* commonly require long breath hold times (~16 s) with minimal respiratory movement. This may be especially difficult in the context of recent acute myocardial infarction, which commonly causes breathlessness and orthopnea.

Alternative methods that can detect IMH with shorter breath hold times are therefore desirable. MR is capable of detecting differences in the magnetic susceptibility of tissues. The paramagnetic properties of hemoglobin products within IMH cause local phase shifts relative to surrounding tissue [8]. The k-space data acquired for each magnitude image can be also used to derive phase data. With the exception of large vessel flow quantification, clinical CMR largely relies on magnitude data, and phase data are mostly discarded. Phase data can be filtered and combined with magnitude data to generate susceptibility weighted MR images (SW MRI) [9]. SW MRI has the inherent potential advantage of short acquisition times, without the need for spin refocusing or multiple images. SW MRI effects may be more pronounced at 3T due to increased phase differences between tissues, whereas myocardial T2 and T2* images may be degraded at higher field strengths, in part due to these susceptibility effects [10].

SW MRI has been used clinically in neuroimaging, to visualize venous structures in the brain [11], and has been shown to be highly sensitive for the detection of cerebral hemorrhage in stroke [12]. We hypothesized that SW MRI could be used to detect hemorrhage following AMI, especially at 3T. We compared the image quality and diagnostic accuracy of SW MRI with T2w and T2* CMR at 3T for the detection of IMH following reperfused AMI.

## Methods

### Patient selection

Patients with first ST-segment elevation AMI, revascularized by primary PCI within 12 hours of onset of pain were prospectively recruited from a single tertiary center from February 2012 to August 2013. AMI was defined as per current guidelines [13]. Exclusion criteria were previous AMI or coronary artery bypass grafting, cardiomyopathy, estimated glomerular filtration rate <30 ml/min/1.73 m^2^, or contraindications to CMR. The study protocol was approved by the institutional research ethics committee (NHS Health Research Authority, NRES Leeds West) and complied with the Declaration of Helsinki; all patients gave written informed consent. Patients with maximal total scar extent (including MO or IMH) less than 2 voxels of the in-plane resolution of LGE (approximately 3–4 mm) were deemed too small for accurate evaluation of the infarct zone and not included in the analysis. Clinical management (including anticoagulation and use of aspiration catheters) was performed blind to the CMR results and at the discretion of the responsible clinician, reflecting contemporary practice and guidelines. All patients were considered for angiotensin converting enzyme inhibitors, beta-blockade, statins, dual antiplatelet therapy and cardiac rehabilitation.

### Image acquisition

All patients had CMR imaging at 3.0 T within 3 days (median 2 days) of their index presentation (Achieva TX, Philips Healthcare, Best, The Netherlands equipped with a Quasar Dual gradient system (40 mT/m; 200 T/m/s) and radiofrequency (RF) shimming with dual-source RF transmission). A dedicated 32-channel cardiac phased array receiver coil was used. Cine imaging was performed using a contiguous stack of parallel short-axis slices covering the whole left ventricle (LV), with a balanced steady-state free precession pulse sequence (echo time (TE) 1.3 ms; repetition time (TR) 2.6 ms; flip angle 40°, spatial resolution 1.6 × 2.0 × 10 mm, 40 phases per cardiac cycle). SW, T2w, T2* and late gadolinium enhancement (LGE) imaging were performed using the ‘3-of-5’ approach by acquiring the central 3 slices of 5 parallel short-axis slices spaced equally from mitral valve annulus to LV apical cap [14]. The same slice geometry, position and a 10 mm slice thickness were used for all pulse sequences. The SW sequence used a black-blood inversion recovery turbo gradient echo sequence (sensitivity encoding (SENSE) parallel acceleration factor 2.3, TR/TE/flip angle 4.1 ms/3.0 ms/20 degrees, spatial resolution 1.8 × 2.5 × 10 mm, typical matrix 212 × 146, pre-pulse black blood delay 775 ms). Magnitude and phase images were generated online at the time of scanning. T2w imaging used a dark-blood T2w fast spin echo short tau inversion-recovery (STIR) sequence (TE 90 ms, TR two R-R intervals, flip angle 90 degrees, spatial resolution 1.7 × 1.7 × 10 mm, typical matrix 208 × 200) and constant level appearance (CLEAR) homogeneity correction. For T2* imaging, 32 gradient echoes were subdivided into six groups, with a linear k-space order within each group contributing to a separate k-space. The echoes used for the center of k-space for each image/group had consistent parity. T2* imaging parameters were as follows: SENSE = 2, TFE factor 8, TR/TE1/echo spacing (ms) 15/2.3/2.2, spatial resolution 1.8 × 2.5 × 10 mm, typical matrix 176 × 128, pre-pulse black blood delay 420 ms, trigger delay set to image in late diastole. To minimize regional myocardial variation for T2* imaging, image-based shimming was employed [10]. 0.1 mmol/kg gadolinium-DTPA (gadopentetate dimeglumine; Magnevist, Bayer, Berlin, Germany) was then administered using a power injector (Spectris, Solaris, PA). LGE imaging was performed at 16–20 minutes following contrast (inversion recovery-prepared T1 weighted gradient echo, inversion time according to Look-Locker scout, TR/TE/flip angle 3.7 ms/2.0 ms/25 degrees, spatial resolution 1.54 × 1.75 × 10 mm, typical matrix 232 × 182). Breath hold times per slice at a typical heart rate of 60/min were: 16 seconds for T2w, 17 seconds for T2* and 4 seconds for SW data acquisition. For each pulse sequence, images with motion or parallel imaging artefact were repeated until any artefact was removed or minimized. The highest quality images were used for analysis.

### Image analysis

Phase and magnitude data were combined into a SW MRI image using the SWIp algorithm (Susceptibility Weighted Imaging with Phase enhancement; previously referred to as “PADRE”) [15,16]. To enable testing of different SWIp parameters, images were processed offline, taking <10 seconds per image (SWIp tool v1.7, Philips Healthcare, The Netherlands). Automated inline processing on the scanner console with fixed parameters is possible with processing time <5 seconds. The SWIp tool calculates a contrast-enhancing mask from the phase images. The phase information can be contaminated by background field effects and so it is initially corrected by applying a homodyne high pass filter to the complex-valued image data [17]. The SWIp phase (φ) mask is defined by a function, the shape of which is controlled by three adjustable parameters, α, β and σ:

$$ Mask(x)=\left\{\begin{array}{cc}\hfill {{\displaystyle {e}^{-\alpha \left(\left|\varphi (x)\right|-\frac{\sigma \ast \pi }{100}\right)}}}^{\beta}\hfill & \hfill if\left|\varphi (x)\right|\ge \frac{\sigma \ast \pi }{100}\hfill \\ {}\hfill 1\hfill & \hfill if\left|\varphi (x)\right|<\frac{\sigma \ast \pi }{100}\hfill \end{array}\right. $$

**Figure 1:**
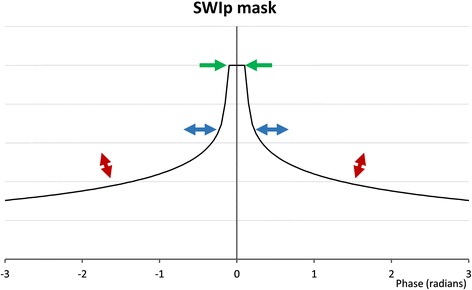
**Effect of parameters on SWIp phase mask.** A plot of mask value (y-axis) vs. phase (x-axis) is shown. α predominantly controls the gradient and height of the mask at higher absolute phase values (red arrows), β predominantly affects the slope of the masking curve at intermediate phase values (blue arrows), and changes in σ predominantly control masking at phase values close to zero (green arrows).

Figure 1 shows the influence of the parameters on the phase mask. The resulting SWIp image is given by the product of the magnitude image and the SWIp mask. As there are no agreed values for α, β or σ for IMH, the effects of varying these parameters were tested on the first 10 patients with visible IMH on T2w imaging. All possible permutations of the following were tested: filter size: 64 × 64, 128 × 128; α: 0, 0.2, 0.4, 0.6, 0.8, 0.95; β: 0.1, 0.2, 0.3, 0.4, 0.55, 0.6, 0.8, 1.0; σ: 0, 0.5, 1, 2, 3, 5, 7.5, 15, 25, 50, 75. These parameter values were chosen to sample the spread of potential values, and also to focus on specific values previously reported [16]. Filter sizes were chosen to account for the larger comparative size of IMH and higher field strength than previous validation work in neuroimaging [18]. Regions of interest (ROI) were drawn using in-house software written in Matlab (Mathworks, Natick, MA, version R2011b) within areas of IMH as defined on T2w imaging and remote myocardium (in myocardium opposite to the infarct zone as defined on LGE imaging and away from the infarct and peri-infarct zone). Areas of hypointensity were visualized and contoured manually. In order to compare different SWIp parameter values, mean signal intensity (SI) and standard deviation (SD) were measured for each myocardial region. The relative signal to noise (rSNR) for IMH regions was evaluated; tissue contrast was evaluated by calculating rSNR difference (ΔrSNR), using the following methods [19]:

$$ rSNR=0.655\times \frac{SI}{SD} $$

$$ \varDelta rSNR=rSN{R}_{\mathrm{remote}}-rSN{R}_{\mathrm{IMH}} $$

The SNR calculation is subject to a number of errors including residual B1 inhomogeneity and non-uniform image noise due to parallel image reconstruction [20]. However, measurement of the absolute SNR was less critical than an accurate estimate of the difference between rSNR measurements. ΔrSNR allows for a quantitative comparison of contrast generation between tissue types in SWIp images using different phase masks, assuming similar coil gain and geometry factor between infarct and IMH ROIs for the acceleration factor used.

The values of filter size, α, β and σ that generated the highest ΔrSNR were derived from the first 10 patients with visible IMH on T2w imaging, and these values then applied to all patients for the main analysis. For analysis, the phase mask was applied once, except where indicated for testing of rSNR and ΔrSNR with multiple applications of the phase mask.

SWIp, T2w and T2* images were independently evaluated for the presence of IMH by two blinded reviewers (AK and DPR, three years of CMR experience). Disagreement between reviewers was resolved by a consensus read. The presented accuracy statistics are based on consensus analysis. A further blinded read was taken (by AK) more than one month after the initial read for intraobserver analysis. The presence of IMH was assessed in conjunction with LGE images, to reflect real-world practice; reviewers were blinded to other imaging sequences, patient and clinical details. IMH was considered to be present when an area of hypointensity was visible within an area corresponding to the infarct zone on LGE imaging. For rSNR analysis, signal intensity and SD were evaluated in IMH and remote myocardial regions based on the consensus read. For T2* images, all echoes were analyzed, the single image per slice with the highest ΔrSNR was chosen for statistical analysis. T2w, T2*, cine and LGE images were evaluated offline using commercial software (cvi42 v4.1.5, Circle Cardiovascular Imaging Inc., Calgary, Canada). Image quality was assessed by consensus of 2 observers, and on a slice-by-slice basis according to a 4 point scale: 4 = excellent, 3 = minor artefact compromising diagnostic accuracy in myocardium outside of the infarct territory, 2 = artefact compromising infarct zone but analysis possible, 1 = unusable. Left ventricular volumes and ejection fraction (EF) were analyzed from cine images using standard methods [21]. Infarct location was determined by LGE imaging, according to standard guidelines [22].

### Statistical analysis

Statistical analysis was performed using IBM SPSS® Statistics 21.0. Continuous variables are expressed as mean ± SD. Normality for quantitative data was established using the Kolmogorov-Smirnov test. Demographic comparisons were performed with an independent samples *t*-test with Bonferroni correction to account for multiple comparisons. Differences in measurements per-slice were evaluated using a multilevel linear mixed-effects model to account for non-independence of slice data. Post-hoc comparisons were made using Tukey’s test. Inter and intra-rater reliability were performed using Cohen’s Kappa statistic. All statistical tests were 2-tailed; p values <0.05 were considered significant.

## Results

54 patients met the inclusion criteria. In 5 patients the infarct size was too small for accurate analysis as per the criteria above; therefore 49 patients were included in the statistical analysis. Patient characteristics are shown in Table 1. Myocardial characteristics are shown in Table 2. No gender-based differences in characteristics were present (p > 0.1 for all).

**Table 1:** **Patient characteristics**

**Patient characteristic**	
n	49
Age, years	58.0 ± 11.3
Male	42 (86%)
Body mass index, kg/m^2^	28.2 ± 3.3
Current smoker	27 (55%)
Hypertension	12 (25%)
Hypercholesterolemia	13 (27%)
Diabetes mellitus	6 (12%)
Pain to balloon time, min (median (IQR^*^))	219 (275)
MO present	25 (51%)
TIMI flow grade ≥ 2 pre-PCI	4 (8%)
TIMI flow grade 3 post PCI	47 (96%)
Peak troponin I, ng/L (median)	>50000
Peak CK^†^, iu/L (median (IQR))	601 (1297)
Infarct territory	
Anterior	22 (45%)
Inferior	21 (43%)
Lateral	6 (12%)

Data as mean ± SD or n (%) unless indicated. ^*^ *IQR* interquartile range, ^†^ *CK* creatine kinase.

**Table 2:** **Infarct characteristics**

**Characteristic**	**Acute visit**
Ejection fraction, %	49 ± 10
LV EDVi^*^, ml/m^2^	82 ± 15
LV ESVi^†^, ml/m^2^	42 ± 12
LV indexed mass, g/m^2^	64 ± 14
LGE infarct volume, ml	48 ± 15
LGE MO volume, ml	3 ± 5
T2w IMH area (per slice, where visible), mm^2^	69 ± 73

n = 49. Data as mean ± SD. LV measurements are indexed to body surface area, infarct volumes are unindexed. ^*^ *LV EDVi* Left ventricular end diastolic volume (indexed), ^†^ *LV ESVi* Left ventricular end systolic volume (indexed).

### Choice of image weighting parameters

The first 10 sequential patients with IMH visible on T2w imaging were selected to evaluate the optimal image weighting parameters for the SWIp sequence to detect IMH. rSNR_IMH_ and ΔrSNR (between IMH and remote myocardium) for each combination of parameters were averaged over the 10 patients. These varied substantially depending on parameter values (rSNR: mean 3.55 ± 0.65, range 1.13–4.28; ΔrSNR mean 0.60 ± 0.25, range 0.00–1.33). The highest and lowest ΔrSNR results with corresponding parameter values are shown in Table 3. The following parameters produced the highest ΔrSNR (i.e. the greatest relative SNR difference between IMH and remote myocardium) and were hence applied to the SW MRI images used for analysis in the whole population: filter size 128 × 128, α = 0.95, β = 0.2, σ = 3.

**Table 3:** **Optimal and worst susceptibility weighting parameters**

	**Filter**	**Alpha**	**Beta**	**Sigma**	**ΔrSNR** ^*^	**rSNR** ^**†**^
1	128	0.95	0.20	3	1.327	2.115
2	64	0.95	0.10	5	1.324	2.025
3	64	0.95	0.20	5	1.321	2.295
4	64	0.80	0.10	5	1.316	2.180
5	128	0.95	0.10	5	1.305	2.702
6	128	0.95	0.30	3	1.304	2.404
7	64	0.95	0.00	7.5	1.301	2.605
8	128	0.80	0.10	3	1.299	1.974
9	64	0.80	0.00	5	1.299	1.904
10	128	0.95	0.10	3	1.297	1.742
1056	128	0.20	0.55	75	0.005	4.019
1055	128	0.80	0.10	0.5	0.008	2.018
1054	64	0.40	0.00	1	0.009	2.528
1053	128	0.95	0.10	0.5	0.028	1.766
1052	64	0.80	0.10	0.5	0.029	2.158
1051	128	0.20	0.55	25	0.033	4.283
1050	64	0.95	0.00	1	0.039	1.270
1049	128	0.20	0.55	7.5	0.043	4.265
1048	128	0.21	0.20	15	0.052	4.255
1047	64	0.80	0.00	1	0.057	1.503

n = 10. Top 10 and bottom 10 values of *ΔrSNR difference between remote myocardium and IMH. ^†^rSNR relative signal to noise ratio of IMH. Lower rSNR values indicate more hypointense IMH.

### rSNR and ΔrSNR

**Figure 2:**
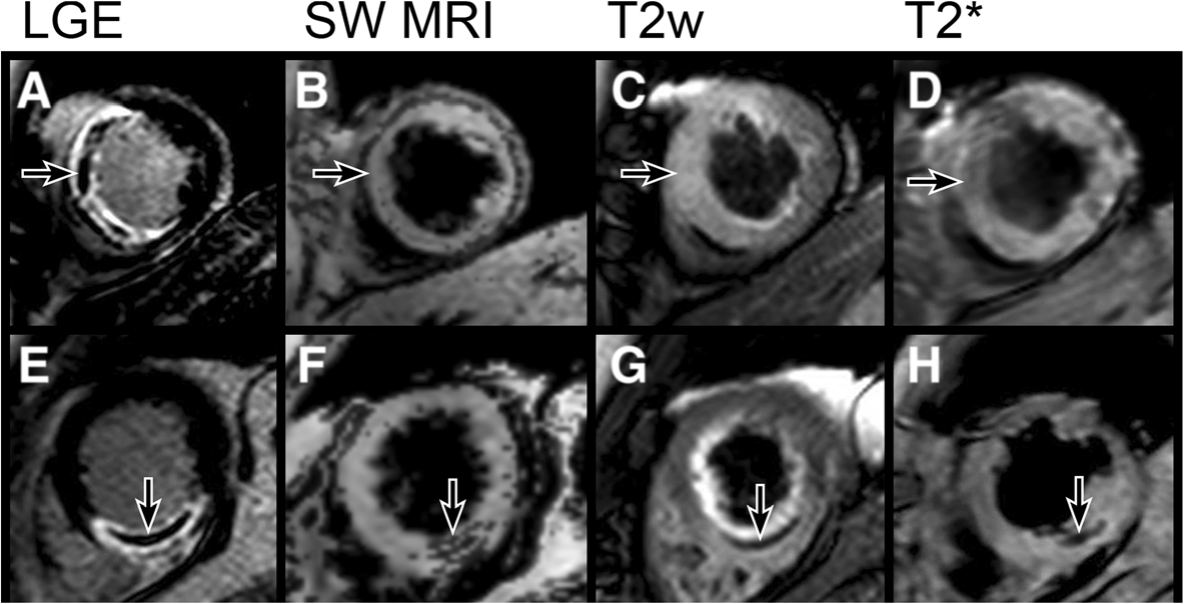
SW MRI comparison with T2w and T2*. Top row: MO as shown by LGE imaging **(A, arrowed)** does not correspond to hypointense myocardium indicating absence of IMH on SW MRI **(B)**, T2w **(C)** and T2* **(D)** images. Bottom row: another patient has MO visible on LGE **(E, arrowed)** corresponding to hypointensity on SW MRI **(F, arrowed)**, T2w **(G, arrowed)** and T2* **(H, arrowed)** images indicating presence of IMH.

**Figure 3:**
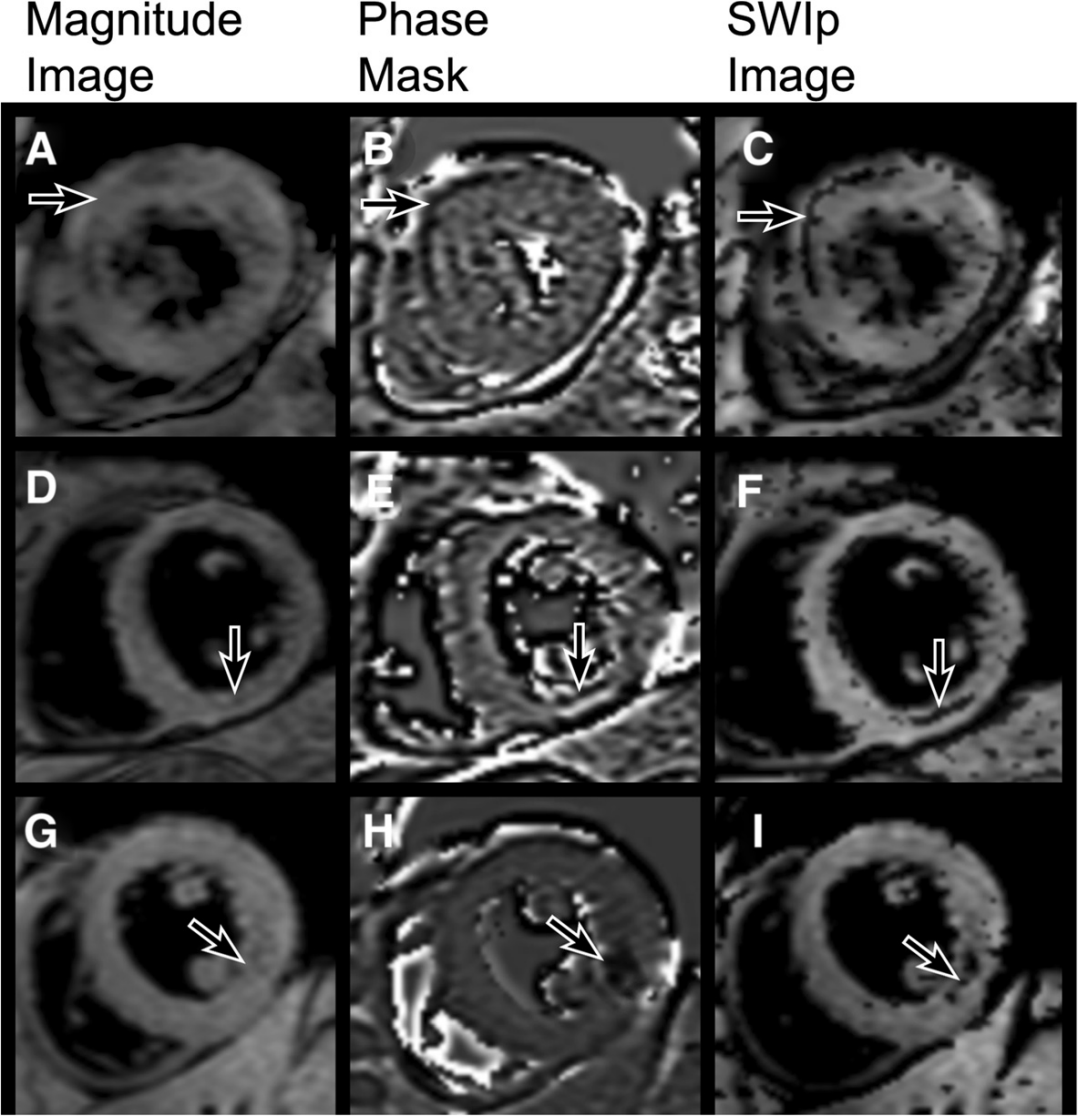
**Contrast generation by SW MRI in three different patients.** Top row: Anterior AMI with IMH (arrowed) is shown in magnitude image without susceptibility weighting **(A)**. A phase mask is generated using the SWIp method **(B)** and applied to the magnitude image to generate SW images with additional contrast for IMH **(C)**. A similar process is shown for IMH in inferior **(D-F)** and inferolateral **(G-I)** territories.

For SW MRI images, average rSNR was 3.62 ± 2.89 for areas of IMH and 5.61 ± 2.63 for remote myocardium (β = 0.47, p < 0.001). ΔrSNR between remote myocardium and IMH was 1.7 ± 2.9. ΔrSNR between infarct and IMH was 4.71 ± 4.48, and between infarct and remote was 3.18 ± 3.41 (β =0.87, p < 0.001). There were no significant differences in SWIp remote rSNR dependent on ROI location (anterior 5.39 ± 3.28, septal 4.16 ± 3.41, inferior 2.46 ± 0.68, lateral 3.33 ± 2.95, β = 0.07, p = 0.39). Figures 2 and 3 show representative images.

For T2w images, average rSNR was 4.23 ± 2.71 for areas of IMH and 4.69 ± 2.04 for remote myocardium (β = 0.00, p = 0.99). ΔrSNR between remote myocardium and IMH was 1.7 ± 2.9. ΔrSNR between infarct and IMH was 2.20 ± 2.27 and between infarct and remote was 1.50 ± 3.59 (β = 0.47, p < 0.01). There were no significant differences in remote rSNR dependent on ROI location (anterior 3.32 ± 1.23, septal 4.64 ± 2.06, inferior 4.60 ± 2.03, lateral 5.53 ± 1.99, β = −0.06, p = 0.46).

T2* images had average rSNR of 5.74 ± 5.81 for areas of IMH and 8.10 ± 4.43 for remote myocardium (β = 0.00, p = 0.99). ΔrSNR between remote myocardium and IMH was 5.35 ± 3.55. ΔrSNR between infarct and IMH was 7.29 ± 3.34 and between infarct and remote was 5.81 ± 3.92 (β = 0.60, p < 0.01). There were no significant differences in remote rSNR dependent on ROI location (anterior 8.50 ± 3.93, septal 9.81 ± 4.68, inferior 8.90 ± 4.45, lateral 7.98 ± 3.58, β = −0.09, p = 0.30).

There was no significant difference between SW MRI and T2w imaging for rSNR in areas of IMH (β = −0.27, p = 0.5), ΔrSNR between remote and IMH (β = −0.19, p = 0.4) and ΔrSNR between infarct and IMH (β = −0.23, p = 0.6). rSNR in IMH was significantly higher in T2* images than SW MRI (β = 0.56, p < 0.01), with no significant difference in ΔrSNR between remote and IMH (β = 0.35, p = 0.1) or ΔrSNR between infarct and IMH (β = −0.69, p = 0.8).

**Figure 4:**
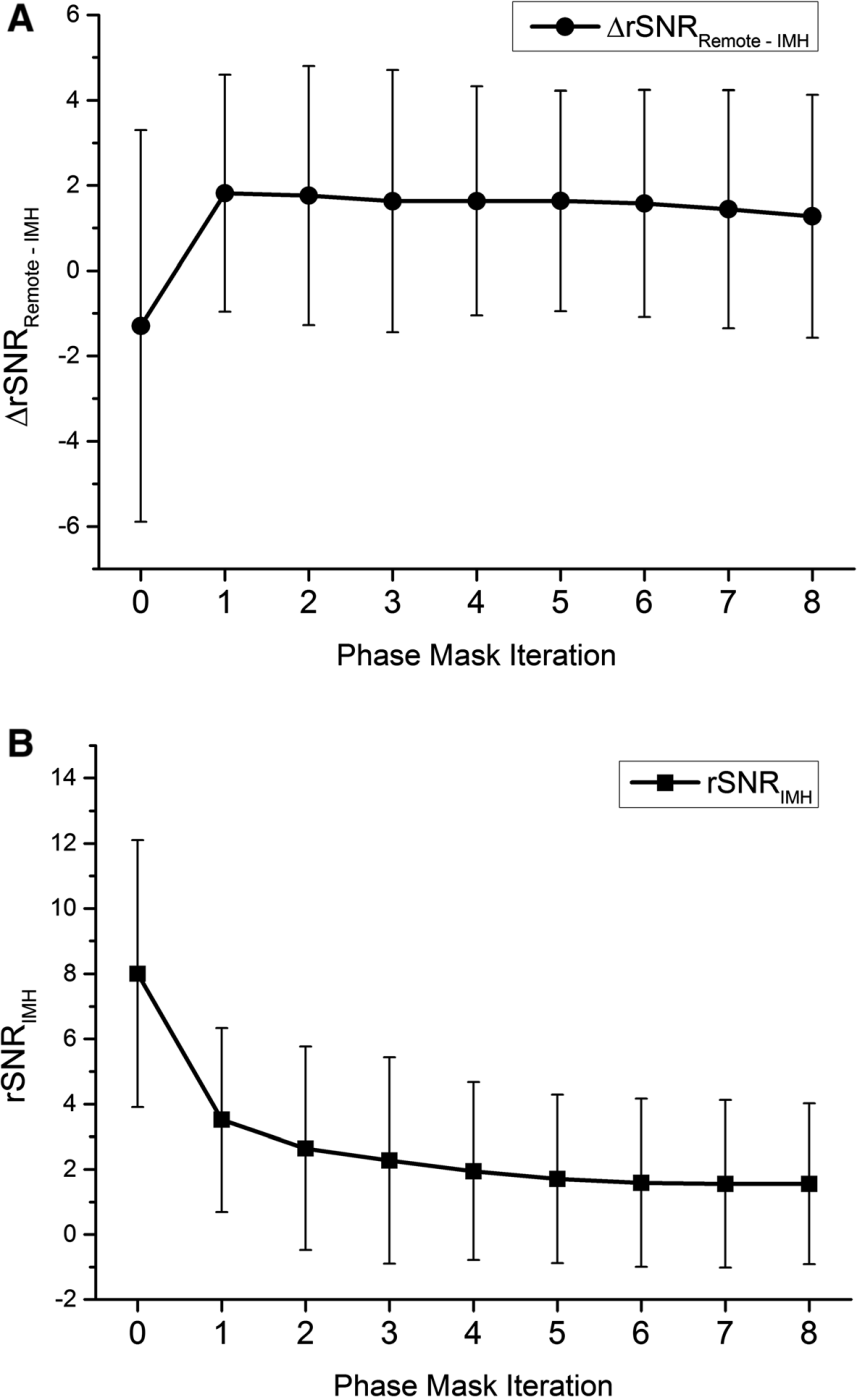
**Effect of successive filtered phase mask applications on ΔrSNR (A) and rSNR of IMH (B).** Values are averaged from all patients with IMH visible on SW MRI (n = 20). Iteration 0 signifies baseline images with 128 × 128 homodyne filter applied but no SWIp phase masking. Error bars indicate SD.

The effect of repeatedly applying the filtered phase mask to the images was evaluated. ΔrSNR and rSNR measurements for each successive iteration of phase mask application are shown in Figure 4. Multiple iterations did not significantly alter ΔrSNR between IMH and infarct over and above the first phase mask application (β = −0.06, p = 0.2, Figure 4a).

### Image quality

**Figure 5:**
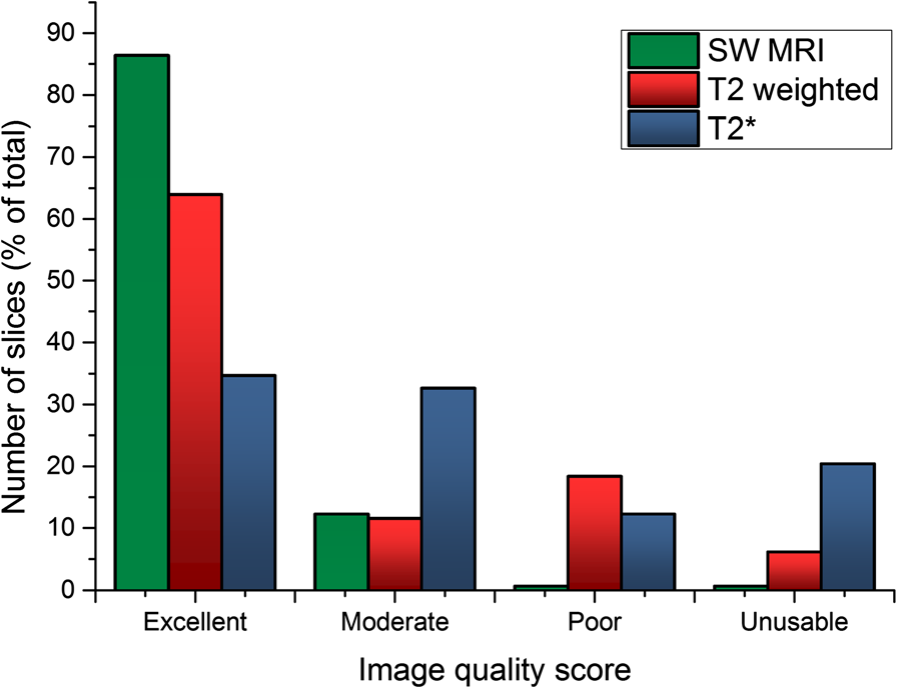
**Per-slice image quality rating.** Images were rated by consensus of two reviewers, blinded to the other sequences, on a scale of 4 (excellent) to 1 (unusable).

**Figure 6:**
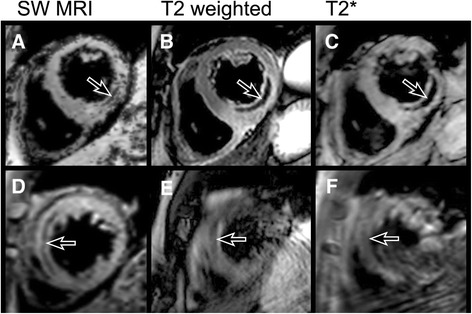
Optimal and suboptimal breath holding in SW MRI, T2w and T2* images. Top row: a patient with good breath holding reveals clear inferior IMH on SW MRI **(A)**, T2w **(B)** and T2* **(C)** images. Bottom row: a different patient with anteroseptal AMI and reduced ability to breath hold clearly demonstrates IMH on SW MRI **(D)**, but T2w **(E)** and T2* **(F)** images are suboptimal to detect IMH.

Mean image quality for SW MRI was 3.8 ± 0.4, for T2w was 3.3 ± 1.0 (p < 0.01 compared to SW MRI) and for T2* was 2.8 ± 1.1 (p < 0.01 compared to SW MRI). One (1%) SW MRI slice, 9 (6%) T2w slices and 30 (20%) T2* slices were graded as unusable (Figure 5). Of the unusable T2w slices, 6 (67%) had clearly visible motion artefact. Figure 6 shows examples of optimal and suboptimal images.

### Image interpretation

Per-slice, IMH within the infarct zone was detected on 34 slices (23%) by SW MRI, 38 slices (26%) by T2w imaging (p = 0.7 compared to SW MRI) and 31 slices (21%) by T2* imaging (p = 0.8 compared to SW MRI). Per-patient detection of IMH was 20 patients (41%) by SW MRI, 21 patients (43%) by T2w (p = 1.0 compared to SW MRI) and 17 (34%) patients by T2* (p = 0.68 compared to SW MRI). Using T2w imaging as the reference standard, SW MRI had sensitivity 93% and specificity 86% on a per-patient basis, and 79% and 96% respectively on a per-slice basis. The inter-observer reliability for detection of IMH by SW MRI was *κ* = 0.82 (95% confidence interval (CI) 0.71 – 0.92), by T2w imaging was *κ* = 0.78 (0.66 – 0.89) and by T2* imaging was *κ* = 0.53 (0.36 – 0.69). Intra-observer reliability was *κ* = 0.79 (0.67 – 0.90), *κ* = 0.79 (0.68 – 0.90) and *κ* = 0.74 (0.61 – 0.87) respectively for SW MRI, T2w and T2* imaging.

## Discussion

This study has found that susceptibility weighted CMR at 3T, using the SWIp technique, can accurately and reproducibly identify areas of intramyocardial hemorrhage following acute myocardial infarction, with superior image quality to T2-weighted and T2* imaging and much shorter breath hold time.

Following reperfusion for AMI, the main clinical utility of CMR is to identify complications that affect patient prognosis. IMH is a strong marker of adverse prognosis, though the most established method of its detection, T2w imaging, is not currently recommended as a routine part of CMR assessment in this context [23]. T2, T2* and SW MRI all rely on the paramagnetic effect of deoxygenated hemoglobin products, which in IMH will also be altered by size of hemorrhage and iron content. T2* imaging is specific for detection of IMH [7], and has been reported to be more robust than T2 imaging at 1.5 T [24]. However, at higher field strengths, increased susceptibility effects and greater B_1_ magnetic field inhomogeneity substantially degrade diagnostic quality [25], whereas these effects, in part, may be utilized to enhance tissue contrast in SW MRI. T2 and T2* imaging are especially difficult post-AMI, as they are sensitive to motion and in general require long breath hold times. Free-breathing T2w and T2* techniques exist [26,27], but rely on technically complicated motion correction algorithms and, unlike SWIp, are not yet available for clinical use. Although T2* appears to have higher contrast when IMH is visible, our data show significantly lower overall image quality for T2* imaging at 3T, and numerically lower detection rates for IMH as compared to SW MRI. In comparison, SW MRI magnitude data have relatively low T2-weighting, and we have shown that by integrating phase data, SW MRI provides comparable diagnostic yield to T2w and T2* with much lower breath hold times (in the order of 4 seconds per slice as compared to 16–17 seconds) and superior image quality.

SW MRI offers a novel method of CMR contrast generation in addition to T1 or T2 relaxation. The technique utilizes the phase data that is acquired with each k-space dataset, but is discarded when producing the magnitude images that are most often used clinically. By processing filtered phase data and combining it with the magnitude image, an anatomical image can be created in which contrast is enhanced by the phase differences produced by deoxygenated blood products (Figure 3) [28]. Phase information can be processed in a number of different ways prior to combining with the magnitude data, and the SWIp technique offers considerable flexibility through the use of multiple parameters, whilst maintaining rapid imaging processing time (<5 seconds for automated inline processing). Neurological applications of SW imaging commonly use TE >10 ms to generate strong T2-weighting, but this would result in unacceptably long shot duration and acquisition time (e.g. 200 ms and 16 s respectively). We have shown that phase data, with appropriate filtering, can be used to help detect hemorrhage in images with relatively short TE and lower T2 weighting, with the benefit of shortened acquisition times and lower image artefacts. In this study we have defined the optimal parameters in this implementation of the SWIp phase mask to enhance IMH.

Susceptibility weighting has been most commonly used in brain imaging, with sparse literature relating to cardiac applications. It is sensitive to early detection of acute hemorrhagic stroke and microbleeds [29], and can also detect intraventricular cerebral hemorrhage in traumatic brain injury and hemorrhagic cerebral tumors [11]. Goldfarb et al. performed an analysis of 11 patients post-AMI, imaged with T2w and SW MRI, and found that phase differences in areas with IMH were significantly different to normal variations in phase difference [30]. The Goldfarb study established the feasibility of the technique, and used a pulse sequence with longer TR and TE, resulting in stronger T2* weighting and relatively long breath hold times. The sequence in the current study deliberately uses shorter echo and repetition times to ensure a shorter breath hold time but with comparable clinical utility to T2w and T2* imaging. Image quality was higher for SW MRI, with an increased proportion of studies without artefact (Figures 5 and 6).

With SWIp, areas of IMH generally often appear as layers rather than a continuous region of reduced signal (Figures 2F and 6A). It is not clear whether this reflects higher spatial discrimination of SW MRI, or whether it is due to the differences between SW MRI and T2w or T2* imaging. Animal studies (such as [31]) typically describe IMH as confluent, macroscopic areas. However, IMH could potentially occur in small volumes of myocardium, below the detection level of T2w or T2* imaging, or tissue may contain heterogeneous areas of IMH. SW MRI may be sensitive to phase changes between small-volume structures and could thus potentially detect smaller areas of IMH, but this clinical study could not test this assumption. Clinically, only the presence and not the size of IMH has been associated with adverse outcome. In the present study, we also did not attempt to quantify the size or severity of IMH, because the amount of signal hypointensity within IMH is related to both iron concentration and oxygenation status of hemoglobin.

Artefact at the ‘heart-lung-liver’ interface at the inferolateral wall was typically much less with SW MRI than with T2* imaging (Figure 6, top row). The optimal parameters derived in this study did result in some aliased pixels in the phase images being carried into the SWIp images. However, these hypointense pixels are clearly distinguishable from true IMH in their small size and position away from the infarct zone (Figure 3).

It has been suggested that multiple phase mask multiplications help to increase the visibility of small areas of hemorrhage in SW brain imaging [9]. We evaluated the impact of multiple SWIp phase mask multiplications on ΔrSNR and rSNR for areas of IMH. In contrast to a previous study [9], we found only a small numerical improvement in ΔrSNR between areas of IMH and infarct between 1 and 2 phase mask applications (Figure 4); however, multiple iterative phase mask applications did not result in a significant improvement in ΔrSNR over the first application.

This study has a number of potential limitations. Other pulse sequences may detect IMH, such as T1 or T2 mapping [24,32], and were not tested in this study; however T2w imaging remains the reference standard with established prognostic utility following AMI [5]. T2w imaging may provide other insights post-AMI, which SW MRI does not, such as estimation of the area at risk and myocardial salvage [33,34]. The SNR of SW MRI, with the mask parameters provided, is likely to change at different field strengths, with the potential to alter diagnostic accuracy.

## Conclusions

SW MRI, using the SWIp technique, is an accurate and reproducible way to detect hemorrhage following AMI at 3T. The technique offers considerably shorter breath hold times to T2w and T2* imaging, and does not appear to be as prone to image artefacts.

## Abbreviations

AMI: Acute myocardial infarction
EF: Ejection fraction
IMH: Intramyocardial hemorrhage
LGE: Late gadolinium enhancement
LV: Left ventricle
MO: Microvascular obstruction
MR: Magnetic resonance
PCI: Percutaneous coronary intervention
RF: Radiofrequency
rSNR: Relative signal to noise
SD: Standard deviation
SW: Susceptibility weighted
SWIp: Susceptibility Weighted Imaging with Phase enhancement
T: Tesla
T2w: T2-weighted
T2*: T2 star
